# Joint sparing treatments in early ankle osteoarthritis: current procedures and future perspectives

**DOI:** 10.1186/s40634-016-0038-4

**Published:** 2016-01-15

**Authors:** Francesco Castagnini, Camilla Pellegrini, Luca Perazzo, Francesca Vannini, Roberto Buda

**Affiliations:** I Clinic of Orthopaedics and Traumatology, Rizzoli Orthopaedic Institute, Bologna, Italy; Orthopaedics and Traumatology, I Clinic, Rizzoli Orthopaedic Institute, University of Bologna, Bologna, Italy

**Keywords:** Ankle, Osteoarthritis, Mesenchymal stem cells, Joint sparing procedures

## Abstract

Ankle osteoarthritis (AOA) is a severe pathology, mostly affecting a post-traumatic young population. Arthroscopic debridement, arthrodiastasis, osteotomy are the current joint sparing procedures, but, in the available studies, controversial results were achieved, with better outcomes in case of limited degeneration. Only osteotomy in case of malalignment is universally accepted as a joint sparing procedure in case of partial AOA. Recently, the biological mechanism of osteoarthritis has been intensively studied: it is a whole joint pathology, affecting cartilage, bone and synovial membrane. In particular, the first stage is characterized by a reversible catabolic activity with a state of chondropenia. Thus, biological procedures for early AOA were proposed in order to delay or to avoid end stage procedures. Mesenchymal stem cells (MSCs) may be a good solution to prevent or reverse degeneration, due to their immunomodulatory features (able to control the catabolic joint environment) and their regenerative osteochondral capabilities (able to treat the chondral defects). In fact, MSCs may regulate the cytokine cascade and the metalloproteinases release, restoring the osteochondral tissue as well. After interesting reports of mesenchymal stem cells seeded on scaffold and applied to cartilage defects in non-degenerated joints, bone marrow derived cells transplantation appears to be a promising technique in order to control the degenerative pathway and restore the osteochondral defects.

## Introduction

Ankle osteoarthritis (AOA) is a severe disabling pathology affecting 1 % of the adult population (Barg et al. 2013; Valderrabano et al. 2009). Primary AOA is usually rare, whereas post-traumatic AOA largely predominates: malleolar fractures and ligament injuries are the most frequent etiologies (Barg et al. 2013; Valderrabano et al. 2009). After acute injuries, there is a conspicuous risk of degenerative progression, ranging from 40 % to 60–70 % in case of complex fractures (Lübbeke et al. 2012). When AOA becomes established, conservative approach could be adopted, but in many cases surgical treatment is needed (Bloch et al. 2015; DiDomenico & Gatalyak 2012). Arthrodesis is still considered the gold standard in case of surgical treatment of severe AOA, but foot joints osteoarthritis may be faced at long follow-up (Bloch et al. 2015; Jordan et al. 2014). Recently, total ankle replacement has gained more and more success, but serious post-operative complications and risks of multiple revision procedures, especially in young patients, are the main disadvantages of the procedure (Bloch et al. 2015; Jordan et al. 2014). Considering the drawbacks of the previous end-stage procedures and the average young age of the patients affected with post traumatic AOA, joint sparing procedures should be considered, mostly in case of precocious degeneration (Bloch et al. 2015; Lübbeke et al. 2012; Madry et al. 2012; Valderrabano et al. 2009). In early degeneration, the whole joint is affected by a mild deteriorating progression, with still a good, reversible balance between anabolic and catabolic processes (Gomoll et al. 2012; Madry et al. 2012). Thus, joint sparing surgical procedures may be desirable, with the aim to delay the degenerative progression, postponing or even obviating the end stage procedures (Gomoll et al. 2012; Madry et al. 2012).

## Review

### Current procedures

Nowadays, three surgical procedures with joint sparing aim are currently performed for early AOA: arthroscopic debridement, arthrodiatasis, osteotomy (Zgonis et al. 2006; Weatherall et al 2013).

### Arthroscopic debridement

Ankle arthroscopy in AOA was reported to be useful in alleviating symptomatology, but ineffective at treating the pathology (Hassouna et al. 2007). In the work by Tol et al, 57 patients with ankle anterior impingement syndrome were arthroscopically treated with debridement (Tol et al. 2001). Patients with no radiographic signs of AOA had excellent outcomes at mid-term follow-up (5–8 years). Patients with AOA had lower outcomes, with a significant relationship with pre-operative radiographic AOA classification: nevertheless, the Authors observed a noticeable clinical improvement. Parma et al described similar outcomes after arthroscopic debridement in patients with anterior impingment (Parma et al. 2014). In a case series of 80 patients analyzed at long-term follow-up (10 years), chondral lesions, advanced age and previous trauma were negative prognostic factors. Patients with narrowing of the joint space had lower outcomes, being the Van Dijk’s classification for AOA predictive of worst results (Tol et al. 2001). Consequently, the Authors proposed a classification of the anterior impingement, mixing the bony spur localization with the cartilage status: patients with diffuse bony impingement and concomitant narrowing of the joint space were doomed to poor results.

Choi et al. analyzed 63 patients with mild or moderate AOA (joint space narrowing) at medium follow-up, after arthroscopic debridement (Choi et al. 2013). Good, but decremental clinical results were reported. Patients with high body mass index and intra-articular lesions, like soft tissue impingement or chondral lesions, had lower outcomes. Hassouna et al reported the results of arthroscopic debridement in 80 patients with ankle impingement or osteoarthritis at mid-term follow-up (Hassouna et al. 2007). In case of AOA, the clinical results were lower, with better outcomes in young patients. At 5 years, 28 % of the patients required a major surgical procedure and 24 % of the cases underwent another arthroscopy: most of them had AOA signs. In conclusion, ankle arthroscopy showed good results at medium follow-up in patients with mild AOA: careful selection of the patients is crucial in order to achieve a satisfying outcome. Arthroscopy cannot be routinely recommended for AOA: otherwise, mild and moderate AOA can be addressed to arthroscopy, adding limited bone marrow stimulation techniques (Zgonis et al. 2006; Weatherall et al 2009) (Table 1).

**Table 1:** Features and results of the selected papers about arthroscopic debridement, arthrodiastasis and osteotomy in AOA

Authors	Year of publication	Type of study	Number of patients	Inclusion criteria	Treatment	Follow-up (months)	Clinical results	Radiological results	Notes
Arthroscopic debridement									
Tol et al	2001	Prospective case series	57	Anterio bony impingement and moderate AOA	Arthroscopic debridement	78	Excellent or good (VAS and Tegner)	65 % successful	AOA reduces the success rate
Parma et al	2014	Retrospective case series	80	Anterio bony impingement and moderate AOA	Arthroscopic debridement	105	Aofas score 70.7 pt at final follow-up	NA	Chondral lesions, age and cavus foot negatively affect the outcome
Choi et al	2013	Retrospective case series	63	Mild and moderate AOA	Arthroscopic debridement	71	Aofas score 76.2 pt at final follow-up	NA	High BMI and chondral lesions reduce the success rate
Hassouna et al	2007	Prospective case series	80	Anterio bony impingement and moderate AOA	Arthroscopic debridement	60	28 % requiring major surgery at 5 years	NA	AOA reduces the success rate
Arthrodiastasis									
Ploegmaker et al	2005	Retrospective case series	25	Severe AOA	Fixed joint distraction	84	73 % clinical benefit at 7 years	NA	Distraction has long term benefit
Marijnissen et al	2002	Open prospective study	57	Severe AOA	Fixed joint distraction	34	Good pain control, more mobility	Joint space width increased 10 %	Improvement increased over the time
Marijnissen et al	2002	Randomized controlled trial	17	Severe AOA	Joint distraction vs debridement	12	Good pain control, more mobility	Less subchondral sclerosis, more joint space	Better clinical and radiological results for arthrodiastasis
Tellisi et al	2009	Retrospective case series	25	Severe AOA	Joint distraction	30	Aofas score at the final follow-up: 74	NA	91 % improved pain
Nguyen et al	2015	Retrospective case series	36	Severe AOA	Fixed joint distraction	60	45 % requiring arthrodesis or replacement	Progression of AOA	Outcome decreased over the time
Marijnissen et al	2014	Retrospective case series	111	Severe AOA	Fixed or hinged joint distraction	144	50 % of failures	NA	Outcome decreased over the time
Osteotomy									
Knupp et al	2011	Prospective case series	94	Asimmetric AOA	Supramalleolar osteotomy	43	Good clinical improvement	AOA improved	10 patients failed
Colin et al	2014	Retrospective case series	83	Asimmetric AOA	Supramalleolar osteotomy	42	Aofas score 73 pt for varus and 80 for valgus	Improved	Sidewalk sign to assess the correct indication
Kim et al	2014	Retrospective case series	31	Asimmetric (varus) AOA	Supramalleolar osteotomy and microfractures	27	Aofas score 83.1 pt at final follow-up	42 % AOA advancement	Microfractures may improve the results

### Arthrodiastasis

Arthrodiastasis is indicated in young patients with AOA and a normal alignment of the ankle joint (Bloch et al. 2015; Kluesner & Wukich 2009). It consists in applying a circular external ring fixator (usually Ilizarov) and performing progressive distraction up to 5 mm. The key of a successful treatment is guaranteeing weight-bearing stability and maintaining the distraction over the time: usually, the treatment should be prolonged to at least 3 months (DiDomenico & Gatalyak 2012; Kluesner & Wukich 2009). Along with the distraction, other associate procedures can be added, like joint debridement, or Achilles tendon lengthening (Bloch et al. 2015; Kluesner & Wukich 2009; Tellisi et al. 2009).

The rationale of this procedure is based on the self-repairing abilities of the osteochondral tissue when unloaded (Intema et al. 2011; Lamm & Gourdine-Shaw 2009). The intermittent intra-articular hydrostatic fluid pressure changes result in a benefit for ankle chondrocytes, which are particularly keen on synthesizing proteoglycans and collagen in response to damage (Intema et al. 2011; Lamm & Gourdine-Shaw 2009). The subchondral bone sclerosis and the bone cysts may be reduced as well, resulting in lower pain and better cartilage repair (Intema et al. 2011; Lamm & Gourdine-Shaw 2009). Lamm et al demonstrated joint space widening with fibrocartilage formation and decreased subchondral bone thickness in MRI after arthrodiastasis (Lamm & Gourdine-Shaw 2009) The main complications of arthrodiastasis are related to infections, neurovascular lesions, hardware failure and damage to ankle ligaments (DiDomenico & Gatalyak 2012). However the main drawback of this technique is linked to patient’s compliance (DiDomenico & Gatalyak 2012).

In the case series with the longest follow-up (7 years), six out of 22 patients treated with ankle distraction failed, requiring arthrodesis or resulting in Sudeck athrophy (Ploegmakers et al. 2005). In the majority of other cases, good and stable clinical outcomes were achieved, resulting in improved ankle range of motion and lower symptomatology over the time (Ploegmakers et al. 2005). In an open study and randomized controlled trial, ankle distraction resulted in good outcomes, with better results over the time (Marijnissen et al. 2002). Radiographic evaluation showed an increase in the joint space, with radiographic improvement over the time. The randomized controlled trial comparing ankle debridement and joint distraction confirmed better clinical and radiographic results for arthrodiastasis. However, about one fourth of the patients did not clinically improve. Tellisi et al described good outcomes as well, reporting only two failures out of 25 patients at short term follow-up (Tellisi et al. 2009). The clinical results improved, with good pain control and better function. In a recent work by Nguyen et al, the clinical and radiological results at mid-term follow-up were not so positive, with 45 % of the patients treated with definitive end stage procedures: differently from the previous studies, the Authors stated that ankle function tended to decline over the time after arthrodiastasis (Nguyen et al. 2015). A similar rate of failure was reported in another study about ankle distraction in AOA at long term follow-up (Marijnissen et al. 2014). Smith et al reviewed the few studies about ankle arthrodiastasis in AOA (Smith et al. 2012). The report described good results, with a small amount of failures at short term follow-up. The Authors highlighted also the weak support by high quality trials (Smith et al. 2012). Nevertheless, the promising results make arthrodiastasis an interesting option for AOA (Table 1).

### Osteotomy

Osteotomy is indicated in case of partial joint degeneration, fracture malunion and axial malalignment, usually traumatic in cause (Zgonis et al. 2006). The rationale of osteotomy is restoring the congruity of the joint and the normal contact areas of the articular surfaces, shifting the forces to an healthy portion of the ankle. Usually a malignment over 10°–15° in any cardinal plane is addressed to osteotomy (Zgonis et al. 2006). Nevertheless, the features and flexibility of hindfoot and midfoot may play a considerable role, compensating the malalignment: usually varus deformity of the ankle is less manageable due to limited eversion abilities of the subtalar joint (DiDomenico & Gatalyak 2012). Contraindication for realignment surgery are end stage, diffuse AOA, infection and neurovascular disorders: the procedure is not recommended even in smokers and older patients with low bone quality (Barg et al. 2013). Osteotomy can be performed around the ankle, in the fibula, or distal tibial metaphysis or diaphyseal junction or in the calcaneus; the deformity to be corrected can be subtalar, supramalleolar or mixed (Myerson & Zide 2013; Zgonis et al. 2006). Many techniques have been described: medial opening or closing wedge osteotomy, corrective Z-shaped osteotomy of the fibula, lateral lengthening calcaneal osteotomy, lateral closing wedge osteotomy (Barg et al. 2013). Frequently, the malalignment has heterogenic etiology, even considering ligament and tendon dysfunction: combined approaches are required to avoid recurrence (Myerson & Zide 2013). The rehabilitation is quite challenging, and sport resumption is recommended not before 4 months (Barg et al. 2013). The results after realignment osteotomy are quite encouraging (Barg et al. 2013). Knupp reported the results of supramalleolar osteotomies in 94 patients after a follow-up of 3 years (Knupp et al. 2011). Only ten ankles failed and required an end-stage procedure (Knupp et al. 2011). Another large case series by Colin et al reported good results in 83 patients treated with supramalleolar osteotomies: seven cases failed after 3,5 years (Colin et al. 2014). Colin et al recognized the clinical sign of the sidewalk (walking on a corrective plane) as positive predictive sign for supramalleolar osteotomy success (Colin et al. 2014). Recently Kim reported the results of supramalleolar osteotomy along with arthroscopic microfractures in the treatment of varus AOA: clinical outcomes after 2 years were encouraging, but tended to worsen over the time (Kim et al. 2014). The radiographic evaluation and the second look arthroscopy showed degenerative progression in 42 % of the cases, with a significative correlation with the clinical outcome (Kim et al. 2014). Realignment procedures are effective, restoring joint congruity and uniform loading, but a correct pre-operative plan must be performed; associate procedures on soft tissues and arthroscopic bone marrow stimulation may improve the results (Table 1).

### Future developments

Osteoarthritis (OA) is characterized by an articular tissue and microenvironment alteration involving all joint components: beyond cartilage, subchondral bone and synovial membrane (Lotz et al. 2014). It has been reported that despite the increase of the number of trabeculae and the bone volume, subchondral bone is hypomineralized and lacks quality due to an abnormal local bone turnover (Li et al. 2013). Moreover, synovitis turned out to play a pivotal role in OA progression, correlating with symptom severity, rate of cartilage degeneration and osteophytosis (Scanzello 2012). So, morphological features of OA include phenotypic changes in chondrocytes, progressive fibrillation of articular cartilage, subchondral bone sclerosis, osteophyte formation, increased remodeling of the periarticular bone and synovial hypertrophy (Li et al. 2013). For this reason, OA is defined a whole joint pathology (Poole 2012).

The early stage of this whole joint pathology is mainly characterized by a catabolic, inflammatory environment and the “chondropenia”: a loss of hyaline articular cartilage volume and biochemical, ultrastructural and mechanical properties modifications of tissue (Aydin et al. 2007; Speziali et al. 2015). Despite the normal gross appearance of cartilage, an altered gene expression profile is already present from the earliest developmental stages of disease (Wang et al. 2009). The progressive degenerative process in OA is caused by an unbalancing of extracellular matrix components turnover as collagens and proteoglycans, in combination with an enhancement of metalloproteinases and aggrecanases (MMP13 and ADAMT-4 and 5) synthesis and secretion (Speziali et al. 2015). Proteolytic enzymes production is induced by proinflammatory citokines (IL-1, NFk-B, TNF-1) which are responsible of the matrix depletion and subsequent cartilage thickness reduction and breakdown (Nöth et al. 2008). Furthermore, OA seems to be associated with changes in the quantity, phenotype, and differentiation potential of resident mesenchymal cells, fundamental for the injury healing process and tissue homeostasis maintaining (Nöth et al. 2008).

The goal of the most straight forwarded surgery for AOA should be the early phase, using a disease modifying, joint sparing procedure with long term effects (Gomoll et al. 2012). So, the ideal treatment should control the catabolic joint environment, reversing the degenerative progress, repairing the osteochondral layer and sparing the native joint.

In case of osteochondral lesions and early signs of AOA, bone marrow stimulation techniques have been usually performed, with quite good results even at long term follow-up (Kim et al. 2014). Nevertheless, the fibrocartilage quality of the new restored osteochondral layer is not desirable, and regenerative technique are usually preferred. However, regenerative procedures has been usually considered a contraindication in case of articular degeneration: the few attempts to implant chondrocytes ended in disappointing results (Filardo et al. 2013; Lee et al. 2012).

Drawbacks of previous treatments along with the potentiality of mesenchymal stem cells (MSCs) make a cell based approach suitable for early AOA. MSCs are multipotent stromal cells that can differentiate in several cell types responding to local environmental stimuli such as cytokines and growth factors, which are released in response of tissue injury (Minguell et al. 2001; Murphy et al. 2013).

Self-renewal and differentiation potentiality are not the unique features of MSCs. MSCs are able to modulate local microenvironment and neighbor cells by cytokines, growth factors, colony stimulating factors and chemokines secretion (Minguell et al. 2001). This paracrine activity is responsible of anti-inflammatory effect of MSCs (Somoza et al. 2014). MSCs produce macromolecules such as collagens, fibronectin, glycosaminoclycans and proteoglycans, important for the extracellular matrix (ECM) organization, a fibrillar network essential in determining tissue architecture by providing a framework for cell adhesion (Nöth et al. 2008).

Another features of MSCs is their immunomodulatory capabilities and the ability of influencing both adaptive and innate immune responses (Zhao et al. 2010).

MSCs have been considered as naturally immunoprivileged cells displaying low expression levels of HLA class I, no expression of HLA class, and no expression of costimulatory molecules (Zhao et al. 2010). It is well-known that under inflammatory stimulation they can express both HLA-I and HLA-II but it is also known that under this condition they exert more potent immunosuppressive actions. The regulatory effect of MSCs on immunological cells has been widely studied demonstrating that, in vivo and in vitro, MSCs inhibit effector T cells proliferation and prevent dendritic cells (DCs) maturation (Spaggiari et al. 2008).

For these reasons, in the last years, MSCs were suggested as a therapeutic tool for the treatment of OA.

Transplantation of bone marrow derived autologous MSCs in patients with knee, hip or ankle OA lacked adverse effects and was completely safe (Emadedin et al. 2015; Jo et al. 2014). Emadedin et al treated 6 moderate and severe AOAs with a single injection of autologous bone marrow derived MSCs, after a previous isolation and expansion process: clinical scores based on pain and functionality increased in the first six months and maintained a positive trend until 30 months (Emadedin et al. 2015). Even MRI showed signs of osteochondral repair and reduction of the subchondral edema. Jo et al reported similar clinical results in 18 knees with moderate or severe OA, treated with a diagnostic arthroscopy and MSCs injection: MRIs and second look arthroscopies with biopsy samples after 6 months demonstrated signs of regeneration, with good filling of the defects and hyaline like cartilage repair (Jo et al. 2014). Moreover, Hauser et al, reported that the direct injection of unfractionated whole bone marrow (WBM) in conjunction with hyperosmotic dextrose for treatment of osteoarthritic joint, reduces pain and improves articular functionality (Hauser & Orlofsky 2013).

In a previous paper by Buda et al, a one-step technique using bone marrow derived cells was applied for osteochondral lesions of the talus (Buda et al. 2015). The procedure employed a concentrate of autologous bone marrow-derived stem cells harvested from the patient’s posterior iliac crest, biological and biodegradable scaffold (hyaluronic acid) and platelet-rich fibrin (PRF), used as a source of growth factors (such as TGF-B1 and PDGF) to improve the healing process. The clinical results achieved a mean excellent Aofas score at 48 months; MRI showed good filling of the defects with well integrated borders in most patients (Mocart scale) and signals compatible with hyaline like cartilage in 85 % of the patients (T2 mapping evaluation). This technique was also compared to a similar cluster of 40 patients treated with autologous chondrocytes implantation: clinical and radiological results were comparable.

The combination of MSCs, biological scaffold and platelet-rich concentrates is fundamental to improve chondrogenic differentiation and cell growth, regeneration of a tissue qualitatively and functionally more similar to normal cartilage, and the control of the articular environment. All of these components generate a bioactive construct completely integrated with the joint microenvironment and surrounding tissues (Schär et al. 2015). This technique should be reserved to focal osteochondral defects and concomitant mild or moderate OA, in young and active patients willing to take on a long rehabilitation.

The principal problem related to the use of native MSCs for transplantation is heterogeneous composition of cell population, including differentiated and undifferentiated cells. To overcome this problem, new strategies for inducing chondrogenic differentiation of MSCs or methods to select a single cell type are under investigation. Application of microRNA (miRNA) and small molecules seem to be an answer since they are able to regulate multiple molecular pathways and cellular processes such as differentiation (Nöth et al. 2008).

## Conclusions

Treatment of early AOA is still debated and no clear guidelines are currently available. Joint sparing procedures are recommended in young patients with AOA, but the results are not so positive and sometimes contradicting: only osteotomy in case of malalignment is universally accepted. Recently, a great interest about biological procedures in early degenerative disease has aroused. Mesenchymal stem cells may play a crucial role in case of degeneration, due to their immunomodulatory peculiarities and the their regenerative prompt: the two features may allow a whole joint approach in case of AOA, regulating the cytokine cascade and restoring the osteochondral tissue as well. Few sporadic data are available about mesenchymal stem cells in degenerated joints, sometimes using an injective procedure and sometimes applying the cells on a scaffold, with encouraging results. In osteochondral defects, few reports about mesenchymal stem cells seeded on scaffold in non-degenerated joints are available: signs of osteochondral regeneration were described. It seems that the autologous bone marrow derived MSCs transplantation may be a promising procedure in order to modulate the degenerative pathway and restore the osteochondral defects.

## References

[CR1] Aydin AT, Ozenci M, Gur S (2007). Chondropenia: early stage degenerative disease. Acta Orthop Traumatol Turc.

[CR2] Barg A, Pagenstert GI, Horisberger M, Paul J, Gloyer M, Henninger HB, Valderrabano V (2013). Supramalleolar osteotomies for degenerative joint disease of the ankle joint: indication, technique and results. Int Orthop.

[CR3] Bloch B, Srinivasan S, Mangwani J (2015). Current Concepts in the Management of Ankle Osteoarthritis: A Systematic Review. J Foot Ankle Surg.

[CR4] Buda R, Vannini F, Castagnini F, Cavallo M, Ruffilli A, Ramponi L, Pagliazzi G, Giannini S (2015). Regenerative treatment in osteochondral lesions of the talus: autologous chondrocyte implantation versus one-step bone marrow derived cells transplantation. Int Orthop.

[CR5] Choi WJ, Choi GW, Kwon HM, Lee JW (2013). Arthroscopic treatment in mild to moderate osteoarthritis of the ankle. Knee Surg Sports Traumatol Arthrosc.

[CR6] Colin F, Gaudot F, Odri G, Judet T (2014). Supramalleolar osteotomy: techniques, indications and outcomes in a series of 83 cases. Orthop Traumatol Surg Res.

[CR7] DiDomenico LA, Gatalyak N (2012). End-stage ankle arthritis: arthrodiastasis, supramalleolar osteotomy, or arthrodesis?. Clin Podiatr Med Surg.

[CR8] Emadedin M, Ghorbani Liastani M, Fazeli R, Mohseni F, Moghadasali R, Mardpour S, Hosseini SE, Niknejadi M, Moeininia F, Aghahossein Fanni A, Baghban Eslaminejhad R, Vosough Dizaji A, Labibzadeh N, Mirazimi Bafghi A, Baharvand H, Aghdami N (2015). Long-Term Follow-up of Intra-articular Injection of Autologous Mesenchymal Stem Cells in Patients with Knee, Ankle, or Hip Osteoarthritis. Arch Iran Med.

[CR9] Filardo G, Vannini F, Marcacci M, Andriolo L, Ferruzzi A, Giannini S, Kon E (2013). Matrix-assisted autologous chondrocyte transplantation for cartilage regeneration in osteoarthritic knees: results and failures at midterm follow-up. Am J Sports Med.

[CR10] Gomoll AH, Filardo G, de Girolamo L, Espregueira-Mendes J, Marcacci M, Rodkey WG, Steadman JR, Zaffagnini S, Kon E (2012). Surgical treatment for early osteoarthritis. Part I: cartilage repair procedures. Knee Surg Sports Traumatol Arthrosc.

[CR11] Hassouna H, Kumar S, Bendall S (2007). Arthroscopic ankle debridement: 5-year survival analysis. Acta Orthop Belg.

[CR12] Hauser RA, Orlofsky A (2013). Regenerative injection therapy with whole bone marrow aspirate for degenerative joint disease: a case series. Clin Med Insights Arthritis Musculoskelet Disord.

[CR13] Intema F, Thomas TP, Anderson DD, Elkins JM, Brown TD, Amendola A, Lafeber FP, Saltzman CL (2011). Subchondral bone remodeling is related to clinical improvement after joint distraction in the treatment of ankle osteoarthritis. Osteoarthritis Cartilage.

[CR14] Kim YS, Park EH, Koh YG, Lee JW (2014). Supramalleolar osteotomy with bone marrow stimulation for varus ankle osteoarthritis: clinical results and second-look arthroscopic evaluation. Am J Sports Med.

[CR15] Kluesner AJ, Wukich DK (2009). Ankle arthrodiastasis. Clin Podiatr Med Surg.

[CR16] Knupp M, Stufkens SA, Bolliger L, Barg A, Hintermann B (2011). Classification and treatment of supramalleolar deformities. Foot Ankle Int.

[CR17] Jo CH, Lee YG, Shin WH, Kim H, Chai JW, Jeong EC, Kim JE, Shim H, Shin JS, Shin IS, Ra JC, Oh S, Yoon KS (2014). Intra-articular injection of mesenchymal stem cells for the treatment of osteoarthritis of the knee: a proof-of-concept clinical trial. Stem Cells.

[CR18] Jordan RW, Chahal GS, Chapman A (2014). Is end-stage ankle arthrosis best managed with total ankle replacement or arthrodesis? A systematic review. Adv Orthop.

[CR19] Lamm BM, Gourdine-Shaw M (2009). MRI evaluation of ankle distraction: a preliminary report. Clin Podiatr Med Surg.

[CR20] Lee KT, Lee YK, Young KW, Park SY, Kim JS (2012). Factors influencing result of autologous chondrocyte implantation in osteochondral lesion of the talus using second look arthroscopy. Scand J Med Sci Sports.

[CR21] Li G, Yin J, Gao J, Cheng TS, Pavlos NJ, Zhang C, Zheng MH (2013). Subchondral bone in osteoarthritis: insight into risk factors and microstructural changes. Arthritis Res Ther.

[CR22] Lotz M, Martel-Pelletier J, Christiansen C, Brandi ML, Bruyère O, Chapurlat R, Collette J, Cooper C, Giacovelli G, Kanis JA, Karsdal MA, Kraus V, Lems WF, Meulenbelt I, Pelletier JP, Raynauld JP, Reiter-Niesert S, Rizzoli R, Sandell LJ, Van Spil WE, Reginster JY (2014). Value of biomarkers in osteoarthritis: current status and perspectives. Postgrad Med J.

[CR23] Lübbeke A, Salvo D, Stern R, Hoffmeyer P, Holzer N, Assal M (2012). Risk factors for post-traumatic osteoarthritis of the ankle: an eighteen year follow-up study. Int Orthop.

[CR24] Madry H, Luyten FP, Facchini A (2012). Biological aspects of early osteoarthritis. Knee Surg Sports Traumatol Arthrosc.

[CR25] Marijnissen AC, Hoekstra MC, Pré BC, van Roermund PM, van Melkebeek J, Amendola A, Maathuis P, Lafeber FP, Welsing PM (2014). Patient characteristics as predictors of clinical outcome of distraction in treatment of severe ankle osteoarthritis. J Orthop Res.

[CR26] Marijnissen AC, Van Roermund PM, Van Melkebeek J, Schenk W, Verbout AJ, Bijlsma JW, Lafeber FP (2002). Clinical benefit of joint distraction in the treatment of severe osteoarthritis of the ankle: proof of concept in an open prospective study and in a randomized controlled study. Arthritis Rheum.

[CR27] Minguell JJ, Erices A, Conget P (2001). Mesenchymal stem cells. Exp Biol Med (Maywood).

[CR28] Murphy MB, Moncivais K, Caplan AI (2013) Mesenchymal stem cells: environmentally responsive therapeutics for regenerative medicine. Exp Mol Med 15;45:e54. doi:10.1038/emm.2013.94. 10.1038/emm.2013.94 PMC3849579 24232253

[CR29] Myerson MS, Zide JR (2013). Management of varus ankle osteoarthritis with joint-preserving osteotomy. Foot Ankle Clin.

[CR30] Nguyen MP, Pedersen DR, Gao Y, Saltzman CL, Amendola A (2015). Intermediate-term follow-up after ankle distraction for treatment of end-stage osteoarthritis. J Bone Joint Surg Am.

[CR31] Nöth U, Steinert AF, Tuan RS (2008). Technology insight: adult mesenchymal stem cells for osteoarthritis therapy. Nat Clin Pract Rheumatol.

[CR32] Parma A, Buda R, Vannini F, Ruffilli A, Cavallo M, Ferruzzi A, Giannini S (2014). Arthroscopic treatment of ankle anterior bony impingement: the long-term clinical outcome. Foot Ankle Int.

[CR33] Ploegmakers JJ, van Roermund PM, van Melkebeek J, Lammens J, Bijlsma JW, Lafeber FP, Marijnissen AC (2005). Prolonged clinical benefit from joint distraction in the treatment of ankle osteoarthritis. Osteoarthritis Cartilage.

[CR34] Poole AR (2012). Osteoarthritis as a whole joint disease. HSS J.

[CR35] Scanzello CR (2012). Pathologic and pathogenic processes in osteoarthritis: the effects of synovitis. HSS J.

[CR36] Schär MO, Diaz-Romero J, Kohl S, Zumstein MA, Nesic D (2015). Platelet-rich concentrates differentially release growth factors and induce cell migration in vitro. Clin Orthop Relat Res.

[CR37] Smith NC, Beaman D, Rozbruch SR, Glazebrook MA (2012). Evidence-based indications for distraction ankle arthroplasty. Foot Ankle Int.

[CR38] Somoza RA, Welter JF, Correa D, Caplan AI (2014). Chondrogenic differentiation of mesenchymal stem cells: challenges and unfulfilled expectations. Tissue Eng Part B Rev.

[CR39] Spaggiari GM, Capobianco A, Abdelrazik H, Becchetti F, Mingari MC, Moretta L (2008). Mesenchymal stem cells inhibit natural killer-cell proliferation, cytotoxicity, and cytokine production: role of indoleamine 2,3-dioxygenase and prostaglandin E2. Blood.

[CR40] Speziali A, Delcogliano M, Tei M, Placella G, Chillemi M, Tiribuzi R, Cerulli G (2015) Chondropenia: current concept review. Musculoskelet Surg 99(3):189-200. doi:10.1007/s12306-015-0377-9. 10.1007/s12306-015-0377-9 26068954

[CR41] Tellisi N, Fragomen AT, Kleinman D, O’Malley MJ, Rozbruch SR (2009). Joint preservation of the osteoarthritic ankle using distraction arthroplasty. Foot Ankle Int.

[CR42] Tol JL, Verheyen CP, van Dijk CN (2001). Arthroscopic treatment of anterior impingement in the ankle. J Bone Joint Surg (Br).

[CR43] Valderrabano V, Horisberger M, Russell I, Dougall H, Hintermann B (2009). Etiology of ankle osteoarthritis. Clin Orthop Relat Res.

[CR44] Wang WZ, Guo X, Duan C, Ma WJ, Zhang YG, Xu P, Gao ZQ, Wang ZF, Yan H, Zhang YF, Yu YX, Chen JC, Lammi MJ (2009). Comparative analysis of gene expression profiles between the normal human cartilage and the one with endemic osteoarthritis. Osteoarthritis Cartilage.

[CR45] Weatherall JM, Mroczek K, McLaurin T, Ding B, Tejwani N (2013). Post-traumatic ankle arthritis. Bull Hosp Jt Dis.

[CR46] Zgonis T, Roukis TS, Polyzois V (2006). Alternatives to ankle implant arthroplasty for posttraumatic ankle arthrosis. Clin Podiatr Med Surg.

[CR47] Zhao S, Wehner R, Bornhäuser M, Wassmuth R, Bachmann M, Schmitz M (2010). Immunomodulatory properties of mesenchymal stromal cells and their therapeutic consequences for immune-mediated disorders. Stem Cells Dev.

